# Collaboration, coordination, and cooperation in occupational health and safety management: perspectives of managers, human resource practitioners, and occupational health and safety representatives

**DOI:** 10.1080/17482631.2025.2563399

**Published:** 2025-09-24

**Authors:** Cathrine Reineholm, Daniel Lundqvist, Christian Ståhl

**Affiliations:** Department of Behavioural Sciences and Learning, Linköping University, Linköping, Sweden

**Keywords:** Work environment, work environment management, occupational safety and health, OSHM, OHSM, qualitative study

## Abstract

**Purpose:**

To increase knowledge about organizations’ OHSM, a specific focus should be placed on how managers, HR practitioners, and OHS representatives conduct OHSM.

**Methods:**

A content analysis was carried out based on semi-structured interviews with 18 managers, 6 HR practitioners, and 5 OHS representatives in different industries and occupations.

**Results:**

All three roles had several OHS assignments. Managers initiate, lead, and check activities; HR practitioners develop routines and guidelines (strategic approach), while OHS representatives often initiate regular safety rounds and inspections (practical approach). The organizational conditions for fulfilling their assignments varied among the three roles. In general, managers and HR practitioners had more favourable conditions, while OHS representatives were dependent on others. Collaboration took place mostly between roles involved in strategic OHSM, i.e., top management and HR practitioners, while cooperation took place between roles involved in operational OHSM, i.e., (first-line) managers and OHS representatives. Regulation-based coordination occurred in a top–down manner through HR practitioners, while reactive coordination occurred in a bottom–up manner through OHS representatives.

**Conclusions:**

For more effective and successful OHSM, more developed interprofessional collaboration is needed between all involved roles. Such collaboration needs to be promoted through improved organizational conditions, regardless of role or organizational level.

## Introduction

Occupational health and safety (OHS) is a central factor for preventing accidents at work, reducing ill health and sick leave (Leka & Jain, 2017), while occupational health and safety management (OHSM) is the activities carried out by an employer to strengthen safety, promote health, and avoid risks in the organization (Reese, 2018). Most countries have legislations that regulate OHS and how organizations should work with OHS issues (International Labour Organization, 2023) or rely on voluntary certifications such as ISO 45001 to improve their work environment practices (ISO, n.d.). However, more knowledge regarding how OHSM is carried out is needed (Zanko & Dawson, 2012). The research that exists focuses mainly on managers (Justesen et al., 2017), e.g., even though OHSM seldom is carried out by one role solely (the manager). Rather, the OHSM is performed by several roles, such as human resource (HR) practitioners and OHS representatives. Given this lack of research, we conducted a qualitative interview study including managers, HR practitioners, and OHS representatives.

The purpose of this study is to increase knowledge about organizations’​​​​​​ OHSM, with a specific focus on how managers, HR practitioners, and OHS representatives conduct OHSM. The following research questions have guided the study:

- What assignments do managers, HR practitioners, and OHS representatives have in the OHSM process?
- How do managers, HR practitioners, and OHS representatives work together in the OHSM process?
- What organizational conditions do managers, HR practitioners, and OHS representatives have to conduct OHSM?

This study contributes to previous knowledge in several ways. A contribution to research is that three different roles in the OHSM are examined and how they work together. Based on this examination of the three roles, an empirically-based model is built that describes how the OHSM is conducted, which holds significance for both research and practice. The study also contributes to practice by clarifying how OHSM can be improved in organizations. For policymakers, the study contributes knowledge that can be used to clarify laws and regulations regarding OHSM.

## Background and literature review

### Context of the study

OHSM is about the activities carried out by an employer with the aim to strengthen safety and health in a workplace and avoiding risks that can harm the health of employees (Reese, 2018). The legislation in Sweden, where this study was carried out, follows the European Framework Directive 89/391/EEC (n.d.). In the Swedish system, the Work Environment Act (n.d.) stipulates that the OHSM must be carried out. In addition, there are several provisions with different focus that prescribe what must be achieved regarding the physical, ergonomic, organizational, and social work environments. A specific provision (AFS 2023:1, n.d.) describes how OHSM is to be carried out. OHSM must take place by employers and employees working together and an OHS representative must exist if there are five employees or more. The OHS representatives must represent the employees and ensure that employers meet statutory requirements. The OHSM must be carried out annually, step by step, in a cyclical process that includes investigation, risk assessment, remediation, and check. There must also be a declaration of intent in the form of an OHS policy as well as documented routines for the OHSM. Assignments in OHSM can be distributed, and if there is a lack of knowledge, such knowledge must be acquired. Although most OHS regulations stipulate that the OHSM must take place (Work Environment Act (SFS 1977:1160), n.d.), more knowledge regarding how organizations work with laws and regulations regarding OHS in practice is needed (Sjö et al., 2022; Zanko & Dawson, 2012).

### Managers, HR practitioners, and OHS representatives in OHSM

In the literature regarding the OHSM, three roles are described as central: the manager, the HR practitioner, and the OHS representative. Most research has focused on managers role in OHSM, showing that the manager is central to how OHSM is carried out (Bergman Bruhn et al., 2023; Justesen et al., 2017; Molin et al., 2021). Despite their important role, studies show that the conditions for managers to implement OHSM are limited. Resources are often insufficient, routines and documentation are missing, and employee commitment is low. Managers also often experience a lack of time for OHSM (Frick, 2014; Hellman et al., 2019; Larsson et al., 2016). In a study on what contributes to managers commitment to OHSM, similar factors are highlighted: time, budget, commitment of senior managers and employees, as well as smooth processes (Tappura et al., 2017). In a systematic literature review, it appears that interventions aimed at managers can result in improved OHSM, improved OHS and health-related outcomes (Sinelnikov et al., 2020).

In textbooks on human resource management (HRM), OHSM is often identified as an essential area for HR practitioners, but empirical research regarding what HR do and what conditions they have to do this is still underdeveloped (Fan et al., 2020; Markoulli et al., 2017; Zanko & Dawson, 2012). A possible explanation could be that HR as a specific professional group is made invisible in studies, as it is often treated as representatives of the employer, or that it is merged with other professional groups, such as OHS professionals or OHS practitioners (Bryson, 2016; Leitã et al., 2017; Olsen, 2012). Research shows, however, that HR practitioners have gone from being a peripheral support unit to becoming an important role in an organization’s OHSM (Schmidt et al., 2017).

An additional central role in OHSM is that of OHS representatives (Friebel et al., 2024; Menendez et al., 2009; Milgate et al., 2002). OHS representatives have several different assignments in OHSM, such as informing and providing consultation on safety issues, participating in risk assessments, annual inspections, and reporting problems to managers or authorities (Brun & Loiselle, 2002; Coulson, 2018; Harris et al., 2012; Milgate et al., 2002). OHS representatives educational background varies, but they tend to have much work experience within their profession (Brun & Loiselle, 2002; Coulson, 2018). One study describes how increasing responsibility for OHS and OHSM is placed on OHS representatives, and in case of incidents, they are blamed (Coulson, 2018).

To carry out efficient and successful OHSM, Milgate et al. (2002) and Blewett and Dorrian (2012) highlight a number of conditions as central: managers commitment, communication, information and training, trade union commitment, internal OHSM systems, OHS committee’s work processes, and OHS professionals (consultants, occupational health services, etc.). Additional conditions that have been identified as central are the organizations size, creditworthiness, safety culture (da Silva & Amaral, 2019; Nordlö et al., 2017), commitment from employees and top management, finance, clarity in what needs to be done, and support systems (da Silva & Amaral, 2019; Mambwe et al., 2021; Savković et al., 2019; Tejamaya et al., 2021).

However, despite calls for a more contextualized picture of those who perform OHS, their roles and existing conditions (Zanko & Dawson, 2012), the research on this topic is still limited. In fact, some studies emphasize that different roles need to work together and support each other to conduct OHSM (Tappura et al., 2014). Other studies suggest that HR practitioners have increasingly assumed a role in the strategic OHSM, leaving operative OHSM to managers. In so doing, the HR department has become a “gatekeeper”, deciding on what OHSM should be carried out, making managers more dependent on their advice (Paulsson et al., 2020; Schmidt et al., 2017).

### Three terms to describe interprofessional collaboration

When different professionals work together in a partnership to benefit or to provide service to a third part (e.g., clients, patients, users), it is often referred to as interprofessional collaboration (Schot et al., 2020). For instance, within the health care sector, physicians, nurses, occupational health therapists, etc., are encouraged to work together, and interprofessional collaboration is established. When different professionals can bridge their professional knowledge and expertise, discuss possible overlaps in roles and work tasks, they can also learn how to handle or cope with factors that hinder collaboration. Interprofessional collaboration is, however, dependent on structural factors and resources, e.g., finance and policies (Schot et al., 2020). The use of the term collaboration can sometimes cause confusion, as it is used synonymously with the terms coordination and cooperation.

In a systematic literature review, Castañ et al. (2020) theoretically investigate and make an attempt to define the meanings of the three terms collaboration, coordination, and cooperation. Coordination refers to deliberating, negotiating, and agreeing on a common goal, while cooperation addresses the implementation of the common goal. Collaboration includes voluntarily helping, supporting, and improving others’ internal performance to achieve the common goal (Castañ et al., 2020). This is also in line with Hammar Chiriac (2014), who differentiated the concepts of cooperation and collaboration. Cooperation refers to a group or a team working together but with no or little interaction, while collaboration includes more interactions and all members competence and skills (Hammar Chiriac, 2014).

Transferred to OHSM, collaboration involves how the different roles help and support each other, bridge their professional knowledge and expertise, and discuss possible overlaps in assignments and responsibilities to achieve common OHS goals. Coordination would entail how they deliberate, negotiate, and agree on common OHS goals, while cooperation would entail the implementation of OHS goals.

## Methods

### Sample and procedure

Interviews were carried out with 18 managers, 6 HR representatives, and 5 OHS representatives (16 women and 13 men) working in different industries and occupations in both the public and private sector (see Table 1). The selection strategy was to obtain a varied sample of managers, HR, and OHS representatives from organizations of different sizes and sectors, representing both blue-collar and white-collar employees. The semi-structured open-ended interview guide developed for this study, included questions about the respondents’ background (position, occupation, experience, etc.), work tasks and responsibilities, OHS and OHSM in their organizations (see Appendix). The interviews were conducted remotely (via Zoom, Teams or over the phone). The interviews lasted approximately 30 min and were recorded and transcribed verbatim. Written and oral information about the study was presented in advance. Before the interview started, additional oral information about the study was given, and informed consent was obtained from the respondents. The respondents were assured that statements were treated confidentially and that they could withdraw from the study at any time.

**Table 1.** Overview over industry, sector, participating managers, HR practitioners, and OHS representatives (*N* = 29).

Industry	Sector	Managers	HR practitioners	OHS representatives
Preschool	Public	1		
Elementary school	Public	1	1	1
Elderly care	Public	1	1	2
Governmental service	Public	2		
Municipal service	Public	2	1	
Fire and rescue	Public	1	1	1
Cultural service	Public	1		
Manufacturing industry	Private	5	2	1
Store	Private	1		
Hotel & restaurant	Private	1		
Recruitment & staffing	Private	2		
Total		18	6	5

### Data analysis

An inductive content analysis (Elo et al., 2008) was performed, based on the three research questions. Initially, the interview transcripts were read in their entirety to obtain a broader understanding of the material. Next, the transcripts were imported into the QSR NVivo software program. Descriptive nodes, derived from the interview questions, were used to obtain a first overall picture of each interview. Examples of descriptive nodes were “Resources”, “Competence”, and “Support”, which were combined into the main theme “Conditions”. The statements were also sorted into managers, HR, and OHS representatives.

## Results

The results section is structured in accordance with the three research questions of the study and begins with the assignments of managers, HR practitioners, and OHS representatives. Next follows how the managers, HR practitioners, and OHS representatives work together, and lastly, how they perceive their organizational conditions to fulfill their OHS assignments.

### Assignment in the OHSM

The interviewed managers were quite aware of their responsibility to initiate, lead and check different OHS activities. The OHS regulations governed the managers work with OHS. These regulations had been transformed in the organization into an annual cycle of activities that most managers based their work on.

According to the interviews, OHS regulations were often initially screened and interpreted at the strategic management levels or HR department and then passed down to the operational management levels for practical interpretation and application. Some managers tried to keep themselves updated on the regulatory changes, while other managers relied on the HR department being in control and provide them with information when regulations were updated.

The managers described that OHS regulations and their application were often discussed and interpreted between managerial colleagues. The managers also helped update each other on regulatory changes. If questions arose or when they did not know what to do, most managers turned to HR for advice. Other (support) organizations, e.g., trade unions, employers, and interest organizations, could also be contacted, often to gain deeper understanding and to ensure that they did the right thing: *"How have you interpreted this? Can you show us? Can you help us?* (Manager 4).

Information about regulations and regulatory changes was also distributed from OHS representatives or from external organizations. Some of the organizations (mainly within the manufacturing industry) had a specific department that dealt with OHS. The OHS department (whose focus was mainly on the physical work environment), informed and educated, and when needed, called for an information meeting: “*And they [OHS department] are very well-read I must say. We have the luxury of being served, which smaller organization don’t get*” (Manager 9).

All interviewed HR practitioners belonged to an HR department, and discussions related to the interpretations of OHS regulations were held with their HR colleagues. These discussions often concern the systematic OHSM and its routines. Regulations concerning the specific organization, for example, regarding chemicals, tools, light, and noise, were also discussed jointly. The HR practitioners described themselves as being responsible for OHS information in the organization, ensuring that managers were updated, maintaining the HR system (which included the internal OHS support system), but also for planning and implementing OHS courses and training. The HR department were often described as a support function, and the HR practitioners interviewed felt they were expected to have good knowledge of OHS and needed to be up-to-date and well-read:

After all, we work as a support function or close business partner, so we’re expected to assist with the competence needed in all matters, and we must always rely on what the legislation says when we give advice and support. Actually, that’s the foundation for what we can recommend. (HR 3)

Almost all HR practitioners agreed that the responsibility for monitoring regulation changes was on HR, as well as updating internal documents and systems. A similar service could be purchased by external companies, who ensured that the organization would keep them updated. In the event of an upcoming change, the HR practitioners went through the change and what it might entail. After that, information was sent out to all managers, sometimes also including a training material:

I expect that we will get support from central HR [department], that information will be released and that we, somehow, spread it to the managers. That is a typical issue that we will raise with the management when we discuss OHS. It comes up as a point [on the agenda], this is a provision that will be changed, a legal requirement. (HR 4)

Several OHS representatives considered themselves as a link between top management, managers, and employees. Although some of the participating organizations had an OHS department dealing with more general and strategic OHS issues, the OHS representatives felt that they were closer to the employees and the daily operations. Ensuring that safety rounds and inspections were carried out regularly, performing risk analyses, and documenting OHSM were tasks that many OHS representatives were responsible for.

### How managers, HR practitioners, and OHS representatives work together

The three roles in the management of OHS teamed up in various ways. Some managers and HR practitioners described that they often worked jointly. Other managers, particularly first-line managers, considered HR more of a support function rather than a partner. The managers contacted HR when a problem had arisen or a new regulation needed to be implemented that the manager did not know how to handle. Similarly, the managers contacted the OHS representatives when a problem or incident had occurred.

The HR practitioners also agreed that a large part of their assignment consisted of supporting managers in OHSM. In large organizations, an HR practitioner could be responsible for an operation or unit, or as a specialist within a specific area, for example, for OHS. In smaller organizations, the HR practitioner had responsibilities in several areas where OHS was one of them. However, supporting managers and explaining how a problem could be solved was sometimes challenging:

It’s a challenge for managers to feel confident, if they have overlooked something in terms of OHS. And it’s a challenge for me as HR to be able to explain and provide information and educate, teach them how to find information. (HR 4)

The OHS representatives were more critical regarding the management of OHS. For instance, the HR department informed the managers regularly regarding OHS issues, but the OHS representatives often felt left out. However, one OHS representative described that teamwork regarding OHS has improved in recent years. From a previous lack of interest in OHS issues, the new HR department showed a great interest and information was now sent out regularly:

Now, we have a HR department that doesn’t have the industry-specific knowledge to a great extent, but a commitment to the organization and well-being, and that we shall follow OHS regulations and so on. So, you’re a bit on your toes, now this has arrived, this will be changed and reviewed and so on. We start to find our way now. Before, we weren’t there, we weren’t there at all. (OHS representative 6)

Although most of the OHS representatives experienced shortcomings in the OHSM, they still felt appreciated by the managers:

I feel that my role has become very important to managers. That there’s support, that there’s someone who books these [safety] rounds, the meetings, what needs to be done every year. .//. And you get the same direction with different departments. So, I think that’s very important. (OHS Representative 5)

### Organizational conditions to conduct OHSM

Most of the managers and HR practitioners generally considered having sufficient organizational conditions for their assignment in OHSM. They felt that the working environment was a prioritized issue in the organization. Some recurring conditions concerned time, finances, competence, and support, which will be elaborated below.

The view of the managers and HR practitioners was not fully shared by the OHS representatives, who instead believed that their conditions for the OHSM were dependent on the manager’s attitude toward OHS. A manager who was interested in and prioritized OHS wanted them to partner up, while a manager who prioritized financial profit over OHS made their work much more difficult. One of the OHS representatives also noted that the commitment of the employees was also important, as the employees needed to report deviations and incidents. The incident reports were described as important tools for measuring OHS progression:

Then, maybe nothing happens at all, but at least they see that something can be counted in. And you get proof that you have reported something, and you can point that out, I reported that two years ago and still you haven’t done anything about it. (OHS representative 2)

Based on the descriptions, however, it was clear that the conditions looked very different between different organizations and industries. As the physical work environment was easier to risk assess, more resources were also devoted to it. An HR practitioner in the manufacturing industry described their OHSM as follows:

If you look at the physical work environment, of course there are resources. It is always on the top of the agenda at all meetings. That says quite a lot. There is no meeting that should not start with “Safety first”. That’s a pretty high focus, I think. The physical environment is about safety, and the idea is that you should go to work and come back in the same condition, and that includes both physically and psychosocially. You shouldn’t go home in a worse condition than you were in when you went to work. (HR 3)

#### Time

Time to plan and conduct OHSM was considered as a critical factor by all respondents. They described that they often had to make time for OHSM, as they often had to prioritize the daily operations.

Overall good conditions, but the daily operation still becomes priority 1. But you have to work with the resources you have, but more time would have been nice. Apart from the fact that we obviously have a tight operational situation, it’s something that we of course understand that we must prioritize and value as important work. (Manager 8)

For managers with a large span of control or responsibility for employees working shifts or nights, making time for OSMs even more difficult. One strategy mentioned to make time for the OHSM was to include it as a fixed topic on workplace meetings. The lack of time for the OHSM was also confirmed by some of the HR practitioners, who tried to make the OHSM easier for managers by developing routines and guidelines for the managers to follow: *“Yes, I could wish there was more time, more resources. There’s a lot that falls on a manager”*. (HR 2)

#### Financial resources

Financial resources were another condition mentioned by the respondents, particularly by managers. The managers described that they often received OHS assignments from the management or HR department, but often without additional financial resources to accomplish these assignments. One example mentioned several times concerned the work premises, work equipment or machines. Several managers mentioned the difficulty of being responsible for the work environment in terms of light, ventilation, electricity, and heating systems, especially when they lacked knowledge about buildings. Requests for investments were often met with resistance, as it would result in increased costs. Replacing work equipment and machines was also met with resistance for the same reasons—increased costs.

A challenge often described by the managers in public organizations was the difficulty of legitimizing personnel care when applying for budget. Because of the financially slimmed organizations, financing personnel care would be made at the expense of the municipal services. One principal explained that personnel care initiatives were non-existent, as it would be at the cost of the pupils:

Looking into our budget in recent years, and I can go back quite far, there’ve been limited additions, which means that if we can’t work out the budget, it’s development initiatives for the employees that you remove, to afford the number of teachers you need in the classroom. (Manager 16)

#### Competence and competence development in OHS

The managers considered themselves to have good knowledge and competence regarding OHS, regulations and OHSM. They admitted their knowledge were more at a general level and that they knew less about specific details. The managers knew how to conduct the daily or ordinary OHSM, and if a situation occurred that they did not know how to handle, they knew where to find more information or turned to the HR department.

I feel that I have that [control] on the major issues, things that I work with on a daily basis. Then the specifics, things that don't happen every day, I probably don't have a clue about that, but then I feel, I have support and seek information myself or ask for help from HR if I need it. (Manager 7)

Several managers attended various courses and trainings regularly, often internal, about OHS and OHS regulations. In addition to formal training, some of the managers, mainly higher/top managers, described that they also discussed and reviewed OHS and OHS regulations on managerial meetings or workshops.

The HR practitioners felt that they were fairly competent regarding OHS and OHS regulations. They described how they often had opportunities for further competence development, and they received the training they wanted and needed. Like the managers did, the HR practitioners felt that their knowledge was more general and did not focus on specific details. On the other hand, they knew where to find information, the scope of the Work Environment Act and its provisions:

Yes, I would say that. I don’t know in detail, but I know where to go in and look it up. It’s quite a large part of our job, to be able to refer. We work quite a lot with labor law issues. (HR 3)

The competence of OHS representatives was described as work experience-based rather than courses or training, and often focused on specific regulations applicable to their workplace or organization rather than on the overall OHSM. Interpreting the OHS regulations was often a struggle, as they lacked specific OHS details or facts. One OHS representative was critical to the OHS training the organization had provided and felt that the quality of the training was too low. Moreover, the top management lacked both competence and interest in OHS:

I work in an organization that doesn’t like to read the provisions nor the Work Environment Act that much, that’s my experience. It is not difficult to become better [at OHS] than several of the senior managers. (OHS representative 6)

#### Support

Support was highlighted as a significant condition by all investigated roles for fulfilling their assignment in the OHSM process. The managers mentioned several sources of support, both formal and informal. Different internal support functions, such as the HR or finance department, was mentioned. Some of the managers also noted the support provided by the OHS representatives. Other external support functions were also mentioned, such as occupational health services, especially in regard to dealing with problems related to stress or psychosocial issues. Informal support was also described as valuable, often provided from different managerial networks or management groups of which the managers were part of.

The HR practitioners often mentioned the support from other HR practitioners at the HR department, and other external sources of support. For instance, in case of major revisions of the OHS provisions, external partners, such as the Swedish Work Environment Agency, could provide help and support. In such cases, representatives from the agency could visit the organization and provide information about the new provision and its purpose, and suggest how to work further.

While several managers mentioned the HR department as a source of support, not all managers did. All three roles interviewed in the study agreed that the HR department had the main responsibility to maintain and update the internal OHS support system and its documents (policies, regulations, instructions, forms, etc.). However, the internal OHS support system was not considered user-friendly nor understandable by the managers. For example, one manager mentioned that it was easier to find answers on Google than in the internal OHS system. The HR practitioners, however, believed that the system was clear and easy to use. The OHS representatives also felt frustrated with the internal OHS system. One OHS representative described it as being complicated and difficult to work in:

It’s a somewhat complicated system. And it takes a long time to get through it, so we're trying to find a rhythm in this now and I'm trying to teach my colleagues how to do it in the systems, and it's not that easy apparently. (OHS Representative 3)

The OHS representatives mostly turned to each other and their trade associations, the Swedish Work Environment Authority’s website or Google, rather than the HR department for support. One of the main reasons was that the HR department only referred them back to the internal digital support system, which they believed did not answer their questions:

We have discussed that for many years, HR has almost disappeared. So, we all have it, everything is in the computer. After all, there are IT systems high and low and you should find it, and there is no help, it’s just a matter of finding the correct IT system to enter. (OHS Representative 5)

The results regarding the managers, HR practitioners, and OHS representatives OHSM assignments, how they work together, and organizational conditions are summarized in Table 2.

**Table 2.** Overview of OHSM assignment, how they work together, and organizational conditions for managers, HR practitioners, and OHS representatives.

	Managers	Human resources	OHS representatives
**Assignment**			
* What*	Responsible to initiate, lead and check OHS.	Support managers OHSM.	Control and watch over OHS in the organization from an employee and daily operation perspective. The link between management, managers, and employees.
* How*	Perform the OHSM annual cycle.	Update and share information, OHS documents and manuals.	Plan and perform risk analyses, safety rounds and inspections.
**Work together**	Some work jointly with HR department and OHS representatives. Others seek support when needed.	A partnership with top management in strategic OHSM. Some provide advice to first-line managers in specific cases.	Some team up with managers; others provide advice or support when needed or in case of problems/incidents
**Organizational conditions**			
* General*	Perceived as sufficient	Perceived as sufficient	Dependent on managers and employees engagement in OHS.
* Time*	Often not enough, owing to prioritization of daily operations.	Often enough, but managers lack of time slows them down.	Often enough time and can thus contribute with their time in the systematic OHSM.
* Financial resources*	Often not enough to complete all assignments.	--	--
* Competence and development*	General competence, especially in daily OHSM. Competence development regularly.	General competence. Competence development regularly.	Applied competence, mainly built on work experience. Few competence development opportunities.
* Support*	Internal and external support: managerial colleagues, HR practitioners, OHS representatives, occupational health care.	Internal and external support: HR colleagues, Swedish work environment agency.	Internal and external support: OHS colleagues, trade organizations.

## Discussion

The purpose of this study was to increase knowledge about organizations OHSM with a specific focus on the assignments of managers, HR practitioners, and OHS representatives in the OHSM process, if and how they work together, and their organizational conditions to conduct OHSM.

The results showed that all roles have several OHSM assignments, but the perceived clarity of their assignments varied. The managers assignments consisted of initiating, leading, and checking the ongoing OHSM activities. The HR practitioners OHSM assignments were usually on a comprehensive and strategic level. Their assignments involved developing routines and guidelines for the organization’s strategic OHSM, but also to guide and support managers in specific OHS cases. The assignments of the OHS representatives were, in comparison, more unclear or undefined. However, they seem to take a more practical approach to OHS activities, e.g., to initiate regular safety rounds and inspections.

Laws and regulations in Sweden clarify the employer’s responsibility for OHS (Work Environment Act (SFS 1977:1160), n.d.). This responsibility is often delegated to managers and would reasonably include both strategic and operative OHSM. However, the results from this study suggest that strategic and operative OHSM are separate processes in the organizations, involving different roles. HR practitioners seem, in general, to take a strategic responsibility in OHSM, while managers maintain the responsibility for implementing and conducting different OHS activities and are thus less strategically involved. This shift in responsibility has also been noted in previous research observing that managers, despite their OHS responsibility, have become increasingly dependent on HR for fulfilling this responsibility (Paulsson et al., 2020; Schmidt et al., 2017). Furthermore, the results show that the OHS representatives have an operative assignment in the OHSM process, in line with previous research (Menendez et al., 2009; Milgate et al., 2002), and that they have limited influence over the strategic OHSM.

The results also show that collaboration, cooperation, and coordination exist between managers, HR practitioners and OHS representatives (Castañ et al., 2020), but all three roles were rarely involved at the same time. The collaboration that took place on a regular basis often involved (top) management and HR practitioners and concerned mostly strategic OHSM. This collaboration seems to focus on translating laws and provisions to the organizational context, and to develop control systems to comply with legal requirements. Cooperation often involves (first-line) managers and OHS representatives and concerns mostly operative OHSM i.e., to implement strategic decisions and to conduct planned and systematic OHSM. This cooperation seems to be focused on investigating, reporting and remedying risks, and identifying OHS issues. Coordination took place regularly and concerned both strategic and operative OHSM. HR practitioners coordinated the strategic OHSM directed towards the managers, while the OHS representatives coordinated the operative OHSM, which was also directed towards the managers. The coordination of the OHSM was thus performed by two of the roles representing two different logics. The first logic comprise strategic activities based on regulations and provisions (top-down process regarding systematic OHSM), while the second logic comprise operative activities based on risks and incidents (bottom-up process regarding reactive OHSM). The roles in the top-down collaboration seem to interact and make use of each other’s competence while the roles in the bottom-up process cooperate and do not use or take advantage of each other’s knowledge and competence (Hammar Chiriac, 2014).

**Figure 1.**
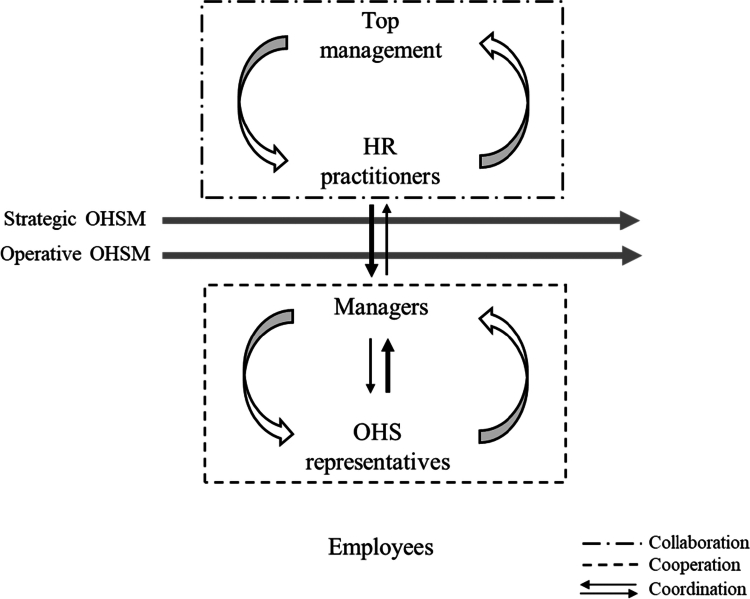
Descriptive model of the collaboration, cooperation and coordination between managers, HR practitioners and OHS representatives in OHSM.

The results of our study further show that interprofessional collaboration exists mostly between HR practitioners and top management and concern the strategic OHSM. This collaboration aims to fulfill regulations, where the HR practitioners assume a leading position in coordinating OHSM towards the managers in the organization. Cooperation occurs between managers and OHS representatives in the operative performance of OHSM In this cooperation, the OHS representatives seem to have taken a leading position, drawing attention to possible risks and incidents in the organization and coordinate OHSM towards the managers. Managers, HR practitioners and OHS representatives and their interactions in the OHSM process are depicted in Figure 1.

The findings also clarify the tension that exists between HR practitioners and OHS representatives, which arises as a result of the different logics their assignments represent, which can be described as systematic versus reactive OHSM. Both HR practitioners and OHS representatives coordinate towards the managers, who are responsible for OHSM and thus face the challenge to unite both perspectives. No bridging seems to occur between HR practitioners and OHS representatives, leaving managers as the spokesperson for both sides. If negotiations take place, they need to be mediated by the managers, making their engagement in OHSM decisive for the process and which OHS issues need to be prioritized.

The organizational conditions for conducting OHSM seem to, in general, be sufficient for fulfilling the OHSM assignment, at least for the managers and the HR practitioners. The OHS representatives were more divided regarding their organizational conditions, referring to their dependency on others engagement in OHSM. Our findings confirm previous studies regarding important organizational conditions for OHSM time, financial resources, competence, and support (da Silva & Amaral, 2019; Mambwe et al., 2021; Savković et al., 2019; Tejamaya et al., 2021). In previous research on the organizational conditions for OHSM, collaboration and cooperation were treated primarily as support. Our study contributes a more nuanced picture of this support, as it is provided by different roles and has different contributions to the OHSM process. However, it is important to note that the organizational conditions are less favourable for OHS representatives than for managers and HR practitioners, e.g., few internal sources for support and less competence development opportunities. Instead, they seem to take more personal responsibility for fulfilling their role in OHSM and external contacts become more important.

Previous research has shown that OHS representatives serve as an important function in the OHSM in organizations (Brun & Loiselle, 2002; Coulson, 2018; Friebel et al., 2024; Harris et al., 2012; Milgate et al., 2002), but our study suggests that their function is not fully utilized. The OHS representatives were seldom invited to participate in the strategic OHSM, even though they probably have the most insight into and knowledge of how work is carried out and the possible risks it might entail. The assignments OHS representatives have in OHSM are at risk of being marginalized, as they are assigned an operative role with less favourable organizational conditions and may only influence the strategic decision-making via the manager, whose involvement is decisive for what should be prioritized. In the long run, this can lead to OHSM becoming a side-activity, a separate process from the main operations (Frick & Walters, 2002), if too much focus is placed on regulation-governed strategic activity. Instead, a balance is needed between strategic and reactive OHSM where OHS representatives play a central role. As suggested by Tappura et al. (2014), successful OHSM require the support and help from different roles. In addition to coordination and cooperation, but also collaboration between the different roles involved in the OHSM process is needed. Interprofessional collaboration (Schot et al., 2020) entails interaction between all three roles as well as the exchange of knowledge and experience, i.e., to bridge professional gaps and to negotiate overlaps in their OHSM roles and assignments. This will, however, be difficult as long as OHS representatives are put aside (e.g., seldom called to meetings, less access to information and internal OHS systems).

### Contribution and implications

This study contributes to research with valuable insights regarding how managers, HR practitioners, and OHS representatives collaborate, cooperate, and coordinate OHSM. Our study also identifies some central organizational conditions for conducting OHSM. A third contribution is the empirically-based model describing how OHSM is conducted in organizations. The model can be used in further research regarding the OHSM as a theoretical starting point, but the model can also be used in practice. Organizations can use the model as a discussion material to improve their OHSM, for instance, to facilitate and to support OHSM collaboration between all OHSM roles, regardless of OHSM assignment and the organizational level. The findings are also relevant for policy makers, as OHSM regulations mainly focus on what needs to be done and not by whom.

Our results suggest that the OHSM is divided into two different processes, one concerning strategic OHSM and one concerning the reactive OHSM. To facilitate a work environment that protects and promotes the health and well-being of employees, it would seem imperative to let these processes mutually influence each other. One way to do this is to ensure interprofessional collaboration between all the roles involved in OHSM.

### Limitations and further research

This study has several limitations that should be addressed. Although we tried to obtain a variety of industries and occupations, the material is not comprehensive. However, there is little to suggest that the assignments, collaboration and conditions of managers, HR practitioners and OHS representatives would differ radically from the results presented in this paper. Both large and medium-sized organizations are represented in the material, while smaller companies are not represented to the same degree. This is due to the study’s purpose to examine all three roles (managers, HR practitioners, and OHS representatives), and HR practitioners are less common in smaller organizations. Another limitation is that gender has not been taken into account, as the focus in the selection process was to include different occupations. Nor did the study consider female- and male-dominated organizations or sectors. The distribution of women and men in the sample, however, is equal. For further research, a gender perspective on the OHSM is thus needed.

## Conclusions

One conclusion of this study is that the OHSM is carried out in two types of parallel processes, one strategic and one operative process. Managers, HR practitioners and OHS representatives have different assignments within these processes, where top management and HR practitioners represent the strategic OHSM while (first-line) managers and OHS representatives represent the operative OHSM. The organizational conditions for fulfilling their assignments vary among these three roles, where managers and HR practitioners generally have more favourable conditions, while OHS representatives are dependent on others, primarily the managers and their work environment priorities. Collaboration seems to take place mostly between the roles involved in strategic OHSM, i.e., top management and HR practitioners, while cooperation seems to take place between the roles involved in operational OHSM, i.e., (first-line) managers and OHS representatives. Regulation-based coordination occurs top–down through HR practitioners, while reactive coordination occurs bottom–up through OHS representatives. For a more effective and successful OHSM, it is argued that a more developed interprofessional collaboration is needed between the different roles involved in the OHSM and that such collaboration needs to be promoted through improved organizational conditions, regardless of role or organizational level.

## Data Availability

The dataset for the current study is held within Linköping University and is available from the corresponding author on reasonable request.

## Appendix 1. Interview guide (word count: 370)

*Background questions*

What is your position in the organization?

Which department/group do you belong to?

How long have you had this position?

How long have you been employed in the organization?

*Occupational health and safety management*

How would you describe the work environment at your workplace?

How do you work with the work environment at your workplace?

Any particular [work environment] tasks or assignments?

Anything specific for the physical work environment?

Anything specific for the psychosocial/organizational work environment?

Have there been any specific investments regarding the work environment in recent years?

Are there any new work environment assignments/tasks/routines etc. that will be introduced soon?

The work that you do with the work environment, what effect does it have?

What significance has the work that is carried out with the work environment for employees’ health and safety?

*Collaboration in work environment management*

Do you work together with someone when it comes to work environment issues?

If so: With whom? How? When?

If you need advice/help/support – who do you turn to?

Do collaboration/cooperation occur between different roles or departments?

If so: Between who? How? When?

External actors?

On whose initiative? Whose responsibility?

*Regulations regarding occupational health and safety*

Do you know what regulations that govern this business/organization/department/workplace relating to the work environment, safety, and health?

How do you implement regulations in your organization/department/workplace?

How does the work with the work environment need to be adapted according to the regulations?

If regulations change, does your work with the work environment change as well? How?

How do you keep yourself updated when it comes to work environment regulations?

*Organizational conditions for occupational health and safety management*

Can you describe your [organizational] conditions for working with the work environment and employees’ health and safety? (e.g., finance, time, support, knowledge/training)

Do you or the organization do something for the work environment that regulations do not require?

Are there any specific [organizational] conditions that you need (more of) to be able to work further with the work environment and employees’ health and safety?

*Finish*

Is there anything that was not covered in the interview that you think is important to add, or something that you want to clarify?

Do you have any questions?
